# Evolving cardiovascular genetic counseling needs in the era of precision medicine

**DOI:** 10.3389/fcvm.2023.1161029

**Published:** 2023-06-23

**Authors:** Ana Morales, Jessica Goehringer, Despina Sanoudou

**Affiliations:** ^1^ Translational Health Sciences Program, School of Medicine and Health Sciences, The George Washington University, Washington, DC, United States; ^2^ Department of Genomic Health, Geisinger, Danville, PA, United States; ^3^ Clinical Genomics and Pharmacogenomics Unit, 4th Department of Internal Medicine, ‘Attikon’ Hospital, Medical School, National and Kapodistrian University of Athens, Athens, Greece; ^4^ Center for New Biotechnologies and Precision Medicine, Medical School, National and Kapodistrian University of Athens, Athens, Greece; ^5^ Molecular Biology Division, Biomedical Research Foundation of the Academy of Athens, Athens, Greece

**Keywords:** cardiovascular disease, genetic disease, genetic counseling, genetic testing, laboratory genetics, personalized medicine, genomics, genomic medicine

## Abstract

In the era of Precision Medicine the approach to disease diagnosis, treatment, and prevention is being transformed across medical specialties, including Cardiology, and increasingly involves genomics approaches. The American Heart Association endorses genetic counseling as an essential component in the successful delivery of cardiovascular genetics care. However, with the dramatic increase in the number of available cardiogenetic tests, the demand, and the test result complexity, there is a need not only for a greater number of genetic counselors but more importantly, for highly specialized cardiovascular genetic counselors. Consequently, there is a pressing need for advanced cardiovascular genetic counseling training, along with innovative online services, telemedicine, and patient-facing digital tools, as the most effective way forward. The speed of implementation of these reforms will be of essence in the translation of scientific advancements into measurable benefits for patients with heritable cardiovascular disease and their families.

## 1. Established genetic testing approaches in CVD

Practicing cardiovascular genetics requires a basic understanding of inheritance patterns. Cardiovascular genetic conditions follow Mendelian inheritance, primarily autosomal dominant (1), although autosomal recessive and X-linked patterns are also observed (2). Specific cardiac conditions that are commonly seen in practice include the cardiomyopathies, arrhythmias, aortopathies, dyslipidemias, and congenital heart defects. Their prevalence and genetic testing yield can range from 1/100 to 1/20,000 and 10%–80%, respectively, depending on the condition. Excellent compilations of each condition's prevalence, cardiac and extra cardiac features, signs, symptoms, and genetic testing yield have been published (Adam MP, Mirzaa GM, Pagon RA, et al., editors. GeneReviews® [Internet]. Seattle (WA): University of Washington, Seattle; 1993–2023. Available at: https://www.ncbi.nlm.nih.gov/books/NBK1116/) (3, 4). It follows that clinical genetic testing and variant interpretation criteria, as established by The American College of Medical Genetics and Genomics (ACMG), are based on the relatively simple monogenic disease framework (5). Until recently, genetic testing in the cardiovascular disease (CVD) clinic involved testing for a single or a handful of genetic variants/genes at a time, with the selection being based largely on clinically informed hypotheses on what the underlying genetic basis, if any, was. Inevitably, this approach led to a large percentage of negative test results, and as a consequence anecdotal evidence indicates that it discouraged, in many instances, clinicians from integrating genetic testing in routine clinical workup. Meanwhile, the cost of these single gene tests was high and the waiting time for the results was often considerably longer than the average genetic tests for other conditions, which in many instances discouraged patients and their families from pursuing it.

## 2. The traditional role of genetic counselors in the CVD clinic

The introduction of genetic testing in the CVD clinics necessitated the introduction of genetic counseling, a patient-centered process that demands the ability to effectively communicate with children and adults of all cultural backgrounds and literacy levels (6). Cardiovascular genetic counselors work hand-in-hand with cardiologists. They collect detailed patient and family medical history, assess risk for cardiovascular disease, and select appropriate genetic tests and communicate guideline-based family screening recommendations. In this process patient ancestry considerations can be valuable (7), since founder variants associated with distinct pathological features are reported for different populations (e.g., *TMEM43* p. S358l in Newfoundlanders; *MYBPC3* c.927-2A > G in Icelanders) (8, 9), and the frequency of certain CVD, such as Brugada syndrome, can vary across populations (10).

Furthermore, at the pre-testing stage cardiovascular genetic counselors provide psychosocial support, discuss genetic testing procedures as appropriate, and obtain informed consent (11). This latter aspect, of informed consent, represents a fundamental part of the genetic counseling process. Psychosocial support is another crucial element of the genetic counseling process. For example, providing anticipatory guidance about concerns arising during genetic testing can help to increase engagement during a process that can be emotionally exhausting for patients and families.

Post-testing, genetic counselors are involved in result interpretation and result disclosure, as well as patient and family support. Once a molecular diagnosis is made in the proband, the genetic counselor proceeds to support cascade genetic testing for relatives. Cascade testing aims to identify those at increased risk who might benefit from surveillance while discharging those who do not. Pre-test counseling for at-risk individuals should include specific discussion items. The collective experience of this author group has been distilled into a suggested genetic counseling agenda as outlined in Table 1.

**Table 1:** Informed consent discussion for cardiovascular genetic testing.

Discussion topic category	Discussion items
**A. Probands**	
Education	• Definition of a genetic test • Testing scope, number and clinical association of genes • Allelic and locus heterogeneity, reduced penetrance • Meaning of a positive, negative or a VUS result • Existing protections and limitations for patient privacy and confidentiality
Genetic testing outcomes	• Probability of positive, negative or VUS result • Possibility of a diagnosis involving extra cardiac features (for example, Fabry disease in hypertrophic cardiomyopathy)
Genetic testing benefits	• Make or confirm a clinical diagnosis • Possibility of treatment and/or management based on molecular diagnosis • Possible option to identify early risk and surveillance options for family members
Genetic testing limitations	• Detection rate for diagnostic testing varies and is not 100% • Some affected individuals express concern about their privacy and risks related to having their genetic information documented in their medical record
Family implications	• Option to pursue cascade testing if proband is positive • Possibility of VUS resolution studies if proband result is uncertain • Potential to reveal genetic status of family members (for example, a parent not pursuing testing may be inferred to be a carrier) • When multiple family members pursue genetic testing, situations of adoption or non-paternity may also be uncovered
Psychosocial support	• Anticipatory guidance about the psychosocial issues that may arise (anxiety, misunderstanding, family issues, and guilt)
Logistics	• Cost • Sample shipment to external laboratory, if applicable • Potential future use of the sample or data after the testing is complete (per genetic testing laboratory's informed consent form) • Preferred time and number to call when results become available
**B. Family members (predictive cascade testing)**	
Education and risk	• Same as proband's (above), except for VUS testing is not offered for predictive testing • Risk of carrying family disease-causing variant
Benefits	• If positive, option for cardiac surveillance • If negative, no need for cardiac surveillance
Limitations	• If positive, it is not possible to predict age of onset or severity
Psychosocial support	• Anticipatory guidance about the psychosocial issues that may arise ◦ Waiting for results (anxiety, uncertainty) ◦ If positive (anxiety, uncertainty about onset or new medical recommendations, cascade risk to relatives) ◦ If negative (survivor's guilt) (Aatre and Day 2011)
Logistics	• Same as proband’s

Genetic counseling aims to facilitate family communication about the option of pursuing testing which can help predict risk of heart disease (12). The complexity of this process grows with multiple disease-causing variants in the proband, an infrequent yet important scenario to be adept at handling. In these situations, risk assessment deviates from the single gene risk figures. In addition, documentation, and financial logistics related to ordering cascade testing for more than one variant must be taken into consideration, which adds time and effort. Cascade testing can be further complicated when family members are geographically scattered. This may necessitate finding a genetics provider local to the family member to facilitate the cascade testing.

When testing at-risk family members for a known disease-causing variant, a negative result can be interpreted with certainty, however, awareness about the value of genetic testing for unaffected at-risk relatives has created new challenges for the genetic counselor. A common presenting challenge is that of an unaffected patient who knows of an affected relative who is otherwise not available for genetic testing. In an individual without evidence of cardiovascular disease but with a family history of genetic heart disease, genetic testing may be an option to consider. However, it's important to interpret the results with care because a negative test result does not necessarily rule out the risk of developing the disease. This scenario requires a careful approach for results interpretation and a dedicated discussion to communicate that a molecular diagnosis can guide cardiac surveillance. While this screening approach is not incorrect, this assessment assumes that the affected relative unavailable for testing carried the same variant. The risk, of course, is that the affected relative had a different diagnosis for which the unaffected patient was not tested.

Similarly, a negative result in this setting is uninformative because the detection rate of diagnostic genetic testing for cardiovascular disease is not 100%. Therefore, it is impossible to determine if the patient tested negative because they did not inherit a variant in a testable gene that would have been detected in the proband, or if the disease-causing variant in the family has not been discovered. In the latter scenario, testing on any family member, affected or not, would be negative.

## 3. New CVD genetic testing tools are more comprehensive and complex

The advent of next generation sequencing (NGS) enabled not only an unprecedented high speed of investigation and discovery of new CVD-related genetic variants, but also the development of new, significantly more advanced genetic testing tools (13). These include “phenotype-specific gene panels”, where tens or hundreds of gene variants associated with different forms of CVD are screened, whole exome sequencing (WES) which assesses almost all protein-coding sequences of an individual, and whole genome sequencing (WGS) which includes nearly all coding and non-coding sequences. Furthermore, polygenic risk scores, the weighted sum of the risk conferred by multiple disease-associated single nucleotide variants (SNVs) across the genome, are gradually introduced into clinical practice aiming at refining risk calculation for CVD and tailoring risk reduction strategies (14).

The introduction of NGS panels radically and permanently changed the complexity and direction of cardiogenetics. With the ability to test for a larger number of genes, novel insights into the genetic architecture of cardiovascular disease, such as the presence of multiple variants in a minority of probands, emerged (15–19). Acknowledging this, variant interpretation criteria have been modified to accommodate the complexity of cardiovascular genetics (20–22). Cardiovascular genetics practitioners have embraced this complexity, and panel testing has become routine.

For example, certain variants in the genes *DSC2*, *PKP2*, and *TMEM43* have a definitive association with arrhythmogenic cardiomyopathy of autosomal dominant inheritance, while variants in the genes *ACTC1*, *MYBPC3*, and *TPM1* have a definitive association with hypertrophic cardiomyopathy (autosomal dominant or recessive forms) (https://search.clinicalgenome.org/kb/gene-validity). Similarly, several definitive gene-disease associations have been established for dilated cardiomyopathy (e.g., *BAG3*, *DES*, and *TTN*), long QT syndrome (e.g., *CALM1*, and *KCNQ1*), and other CVD (https://search.clinicalgenome.org/kb/gene-validity). However, different variants in a gene can have very different effects at the molecular and clinical level, while in many instances variable expressivity and reduced penetrance for a given gene variant is observed. For example, over 170 variants have been reported in *MYH7*, with 29 being classified as pathogenic. Some of those have been associated with hypertrophic, dilated or arrhythmogenic cardiomyopathy, and in some cases incomplete penetrance has been reported (e.g., *MYH7* Arg870His) (https://erepo.clinicalgenome.org/evrepo/). In other instances, such as in the case of *PLN* R14del, incomplete penetrance and a highly variable clinical phenotype ranging from normal to heart failure have been reported, with extensive ongoing research work aiming at deciphering pathogenesis (23–26). However, for many gene variants their association to CVD is moderate, weak, or unknown.

The Clinical Genome Resource (ClinGen) was established by the US National Institute of Health (NIH), primarily funded by the National Human Genome Research Institute (NHGRI), to aggregate relevant data about genes, genetic conditions, and the genetic variants that cause them, and curate them towards the establishment of a public resource of clinically relevant genes and variants to support both the clinical and research settings (https://www.clinicalgenome.org/). Through the close collaboration of experts from a broad range of different fields, including genetics, medical, informatics, academia, and industry, valuable tools for determining gene-disease validity, dosage sensitivity, variant pathogenicity and clinical actionability are being determined. The continuous enrichment of ClinGen and possibly other large-scale international clinical genetic resources, offers the means for fast, easy and centralized access to the latest scientific evidence that has been curated by expert panels, with consistent widely agreed criteria (27).

Clinically available panels can target all cardiogenetic conditions (“pancardio” panels), specific clinical areas (e.g., dyslipidemia, cardiomyopathy, arrhythmia, connective tissue disorders, congenital heart disease), or specific phenotypes (e.g., hypertrophic cardiomyopathy, catecholaminergic polyventricular tachycardia, hypertriglyceridemia, heterotaxy). Genetic testing targeting the appropriate set of genes ensures that results have the highest possible degree of informativeness while decreasing the number of variants of uncertain significance (VUS) or other irrelevant results. In its review of cardiogenetics guidelines and statements, the American Heart Association (AHA) endorsed the genes that should be considered during a genetic evaluation of patients presenting with specific phenotypes (28). In addition, to minimize misunderstanding about the clinical significance of weak gene-disease associations or VUS, guidelines recommend that clinicians use judgment when choosing the number of genes to test (28). While phenotype-specific panels (e.g., hypertrophic cardiomyopathy) have proven value, genetic testing for cardiomyopathies and arrhythmias may benefit from a broad panel approach, as shown by a recent study evaluating the utility of a combined cardiomyopathy-arrhythmia panel. The study reported that ∼11% of molecular diagnoses would have been missed if genetic testing only targeted the genes associated with the patient's phenotype. Furthermore, 36% of these molecular diagnoses that would have been missed included a cardiomyopathy positive result in patients with an arrhythmia indication and vice versa (29). Patients may also present with an atypical personal or family history that, in addition or in substitution of clinical genetic testing may warrant consideration of research genetic testing targeting novel genes or complex genomic regions in known genes. One of the most significant contributions of genetic counselors in clinical practice is choosing the most appropriate genetic test (11). Research and commercial laboratories voluntarily submit information about their available genetic tests to the Genetic Testing Registry (GTR), a database that includes genes tested, methodology, and contact information for laboratories around the world (30). Genetic counselors often refer to the GTR to identify available tests, confirm indication for testing, and compare quality metrics across laboratories.

The results of these tests and their interpretation are significantly more complex than those of previous genetic test generations. Although some genetic variants have been directly linked to specific CVD, many others may have partial, indirect, disease modifying, or unknown roles. In each of these cases a personalized genetic counseling approach that includes careful consideration of the clinical findings and the family history is required (31, 32). Another challenge associated with genomic testing is reporting multiple variants or secondary findings that may have been detected on expanded panel tests, WES, or WGS. This is especially complex when the combined effect of multiple variants is unknown. Furthermore, there is mounting evidence that integrating polygenic risk scores (PRS) into clinical care for CVD can enhance disease prevention and treatment (33, 34). As PRSs evolve, knowledge of when and how to use them in CVD will require appropriate training.

For the VUS in specific, extra caution in the communication of the results is warranted, and a long-term follow-up strategy needs to be implemented, especially in pediatric populations (13, 35). Vague documentation of the findings, limited post-test counseling and poor understanding by the family can lead to false impressions of a molecularly confirmed diagnosis. Meanwhile, as our understanding of the role of different genetic variants expands, currently inconclusive results need to be re-evaluated periodically, and when possible, used to draw new predictive or diagnostic conclusions. Genetic counselors are perfectly positioned to fill this role, ensuring the timely, appropriate, and effective communication of the new information with the carriers and/or their families, along with providing the necessary support towards managing this information.

Beyond diagnostic and cascade testing, an increasing number of healthy individuals seek genetic testing out of concern for a known risk in the family. Meanwhile, others may be unaware of a genotype that puts them at risk for heart issues that may be unmasked upon exposure to stressors, such as pregnancy or athletic training. These scenarios provide a rationale for the idea of population genetic screening for cardiac disease, which has been discussed for some time now (36). New initiatives have rolled out, supporting population screening for familial hypercholesterolemia (FH) (37, 38).

Recent evidence supports the utility of wellness cardiac testing. For example, in a study of 10,478 individuals who underwent genetic screening, 608 (5.8%) had variants identified in cardiac genes, including those associated with arrhythmia, aortopathy, cardiomyopathy, and FH (39). This concept is not entirely new, as it has been practiced for carrier screening. Furthermore, the ACMG recommendations for secondary findings reporting is a nod to the idea that pathogenic variants exist in the population that may not be identified without genetic screening (40). A study of 900 biobank participants tested for ACMG secondary findings genes is aligned with this concept, with pathogenic or likely pathogenic variants in cardiac genes identified in 2%. The study also found evidence of cardiac disease in their electronic medical record, supporting the utility of this approach (41). The implementation of genetic screening programs for heart disease risk should be evaluated with caution. For example, screening athletes for heart disease using genetic testing has been debated. Current consensus does not seem to support blind genetic screening of athletes. Instead, experts recommend that athletes should only be genetically screened in the setting of a suspicious cardiogenetics phenotype or with a positive family history (42).

## 4. The frequency of genetic testing is rapidly increasing

The significant technological advances have enabled faster and expanded analysis, at a fraction of the original cost, along with improved data analysis and interpretation abilities (43–45). Together with increased awareness across healthcare professionals and public interest, this established the vision and groundwork for widespread genomic testing enabling more people to have testing now than ever before.

Driven by phenotype and family history, single gene testing, panel testing, WES or WGS are increasingly being ordered clinically as genetic testing options continue to expand. In 2020 there were 166,703 genetic tests available for ordering (46). Genomic testing is also rapidly increasing in the research arena as a result of large-scale precision medicine initiatives. National cohorts including the All of US Research Program, the Million Veteran Program, the UK Biobank, and FinnGen have aggregated enormous amounts of genomic, laboratory, environmental, lifestyle inventories, and follow-up metrics (47). More recently, Africa announced the “Three Million African Genomes (3MAG),” a sequencing endeavor expected to be developed over the next decade (48). Numerous academic and research institutions are also undertaking precision medicine research. With more research comes knowledge and insight into genomic variability, which has the potential to lead to clinical, generalized population screening for actionable variants in the future.

Meanwhile, a growing number of individuals worldwide receive genetic test results outside of the purview of their healthcare team or research (49). An article in the MIT Technology Review reports that by 2019, over 26 million people across the globe had genetic testing by one of the four leading direct-to-consumer companies (50). Also, genetic testing industry leaders are partnering with employers to expand access to genetic testing towards improving population health. The Stakeholders Assessing Genetics with Employers (SAGE) project sought to characterize the current state of employer-sponsored genetic testing in the context of wellness programs in the US (51). These results showed that, while genetic testing is in its infancy in terms of market adoption by employers, some employers are interested in precision medicine initiatives to improve employee health outcomes.

## 5. The rapid developments in CVD genetic testing pose significant challenges to healthcare professionals and patients

Anecdotally, leaders in cardiogenetics describe the latest developments and the future ahead as exciting, while mainstream providers still grapple with the ever-changing landscape of this field. Illustrating this point, a recent survey of cardiologists and electrophysiologists showed that cardiogenetics providers are not confident about ordering genetic testing or leading appropriate follow-up, expressing the need for more education in cardiogenetics (52). Previous studies have reported similar findings. For example, in a Dutch survey of cardiologists, it was found that the level of experience and self-reported knowledge was low (53). Another study of Swedish healthcare providers showed insufficient knowledge about hypertrophic cardiomyopathy, which the authors identified as a factor interfering with guideline implementation (54).

Cardiogenetic conditions have reduced penetrance, variable expressivity and marked allelic and locus heterogeneity. These factors complicate variant interpretation because of the challenges in identifying proper case, control and family data to feed lines of molecular evidence. Because of this, interpretation discrepancies occur (55). Most concerning is results' misinterpretation, which has led to inappropriate device placement (56). Uninformative results can not only obfuscate the clinician but can also be a source of confusion for the patient (57, 58). Moreover, as variant interpretation understanding evolves, updated laboratory reports are warranted, which often leads to changes in clinical recommendations (59, 60).

The AHA has therefore taken initiative to combat the lack of education, which has kept cardiogenetic testing underutilized (61, 62). They have recommended that cardiologists develop competencies in cardiogenetics (63) and have advocated for the establishment of formal education programs in cardiogenetics (63, 64).

For patients, the thought or pursuit of genetic testing can trigger a broad range of emotions including anxiety, fear, privacy concerns, shame, and confusion, while the risk of misunderstanding the results and making inappropriate medical or daily life decisions is high in the absence of specialist healthcare provider guidance (11, 65).

In light of these challenges, the AHA endorses genetic counseling as an essential component in the successful delivery of cardiovascular genetics care (28, 64), and, from the patient perspective, several lines of evidence support this view. Empowerment, a patient-reported outcome of multiple dimensions, including emotional regulation, hope, and decisional, cognitive, and behavioral control (66–68), has been demonstrated with the intervention of cardiovascular genetic counseling by use of the Genetic Counseling Outcome Scale (GCOS). Through pre- and post-genetic counseling surveys leveraging this scale, a study of patients seen at a cardiovascular genetics center demonstrated a significant increase in empowerment (69). In another study of patients with arrhythmogenic cardiomyopathy, empowerment also increased with genetic counseling, explained by the strength of the patient-genetic counselor relationship, not by the event of ordering a genetic test (70). Reduced anxiety has been demonstrated with the intervention of cardiovascular genetic counseling (71), as well as high patient satisfaction (72), even in comparison to follow up provided by a cardiologist (73).

## 6. The genetic counseling needs in CVD are evolving

Along with the dramatic increase in the number of available cardiogenetic tests, the increasing test result complexity, and demand, there is a need not only for a greater number of genetic counselors but importantly, for highly specialized cardiovascular genetic counselors that will be continuously updated on the developments of the field (11).

This need for an increase in the training and availability of cardiovascular genetic counselors is made apparent when looking at the literature. The 2022 National Society of Genetic Counselors (NSGC) Professional Status Survey ascertained the percentage of genetic counselors who specialize in various areas of practice in the United States (74). Only 3% (*N* = 95) of all respondents (*N* = 2,740) selected cardiology as their primary area of practice. A formal workforce study conducted by the 2015 Genetic Counselor Workforce Working Group indicated a shortage of clinical GCs in North America (across all areas of specialty) and concluded that supply may not reach equilibrium until 2030 (75). Arguably, the situation has been aggravated further by the accelerated speed of new cardiogenetics testing development. Separate from the need of more genetic counselors graduating into the workforce is the need for healthcare institutions to hire enough genetic counselors in needed specialty areas to meet patient needs. This is impacted by the on-going initiative in the U.S. through the Access to Genetic Counselor Services Act (H.R. 2144/S.1450) to pass federal legislation that would permit the Centers for Medicare and Medicaid Services (CMS) to recognize genetic counselors as providers, thereby allowing them to bill CMS for services rendered (76). Though genetic counselors are generally considered to provide a cost-saving service to patients, lack of recognition by CMS impacts the ability for institutions to hire and retain genetic counselors due to limited billing and reimbursement (77). A similar struggle exists in parts of the world with Lynch and Borg (78) showing there is a wide disparity of staffing levels in clinical genetics units throughout Europe. Workforce shortages, insufficient training, lack of experience in handling results from complex cardiogenetic tests, or, in some cases, lack of access to specialized cardiogenetic counseling necessitate the development of an all-encompassing strategy by the international Cardiovascular Healthcare community.

### 6.1. Strengthening the existing genetic counseling model

While an evaluation of how genetic counseling training programs are addressing the challenges outlined is out of scope for this review, it is noteworthy that many programs are taking measures to address these issues. It is essential to recognize that challenges surrounding cardiovascular genetic counseling are applicable to other subspecialties. Training programs are on the right track to overcome these crucial challenges. Meanwhile, despite AHA recommendations to incorporate genetic counseling and genetic counselors into cardiology practice, cardiogenetic testing remains underutilized. Service delivery models integrating cardiologists and genetic counselors have been successfully implemented, with collaboration between genetic counselors and general cardiologists most often reported (79). Still, with heart disease being the leading cause of death in the US with over 650,000 deaths per year (80) and an estimated prevalence of cardiogenetic conditions in more than 1 in 110 individuals (10, 81–84), 3% is an insufficient number of cardiovascular genetic counselors. These figures are especially concerning when the first and only sign of cardiogenetic disease can be sudden cardiac death (85). More genetic counselors with the clinical acumen to effectively support cardiologists, patients, and their families are needed now.

Increase in the capacity of ongoing training programs, establishment of new ones, and creating flexible opportunities for certification (including an on-demand option) are some of the steps that could help combat the genetic counselor shortage and address the evolving clinical needs of CVD patients in a timely manner. At the writing of this paper, there were 54 accredited genetic counseling training programs and six emerging programs in various application stages in the U.S. (86), speaking to the effort to increase the number of genetic counselors graduating annually. Additionally, undergraduate colleges and universities are introducing genetic counseling certificate programs in an effort to expose students to genetic counseling, attempting to generate interest in this field (89). Careful consideration, coordination and regulation at the national and international levels will be needed to ensure that the highest training standards are maintained and the numbers of newly certified genetic counselors are finely adjusted to current and anticipated future needs.

The harmonization across established programs, as well as new genetic counseling training curricula and certification processes internationally is important for the high-level clinical care of CVD patients. Parameters adding to this necessity include the increasingly available options to patients for cross-border genetic testing, genetic testing at different laboratories or different times, communication with multiple genetic counselors at the same or different locations over time, as well as genetic counselor mobility and online genetic counseling services.

In some countries, addressing the national genetic counseling needs may require establishing genetic counseling as a new allied health profession, along with genetic counseling specialty programs and formal certification procedures. These efforts can capitalize on the existing, strong foundations set by well-established curricula and experienced genetic counselors, while learning from reported shortcomings or challenges historically faced. However, the steps towards this direction can be strikingly slow and challenging in certain countries/cultures where long-standing practices can hold back the advancement of clinical practice. International genetic counseling awareness campaigns targeting health authorities, healthcare professionals, patient groups and lay public in those countries, along with closer collaboration of internationally certified genetic counselors with local healthcare professionals and patient groups could be instrumental towards this end.

### 6.2. Capitalizing on technological advancements

Another strategy to meet patient needs involves harnessing service delivery models beyond the traditional, scheduled in-person care. Many medical institutions and insurance companies in the US offer online, immediate access to providers for both urgent and non-urgent indications. This trend for rapid or on-demand service is starting to permeate genetics care delivery.

Telehealth is another example of a service delivery model that has the potential to improve access to genetic counselors. For several years companies in the US have helped fill a gap in the services within certain institutions and geographic regions by offering a telehealth genetic counseling model. Further, the COVID-19 pandemic increased access to telehealth services, which in turn increased the amount of patients providers could care for (90), without jeopardizing patient satisfaction (91). Telehealth approaches could also be a complementary solution for regions without any or sufficient access to genetics services. As of the date this manuscript was written, the NSGC directory findageneticcounselor.com (92) listed 148 and 103 cardiovascular genetic counselors offering in-person and telehealth consults, respectively, a commendable effort to continue promoting access to this much needed service.

Similarly, digital genetics tools are increasingly being developed to meet the need for patient education and care, and could improve genetics service delivery by overcoming geographical and time constraints. Lee et al. (93) identified and analyzed 70 studies that explored unique patient-facing digital tools (any digital tool that is intended for use by patients) used in genetics service delivery. The great majority (84%) of these studies reported positive patient outcomes, and improved workflow and work efficiency. Patient-facing CVD digital tools are slowly emerging. A recent study addressed at-risk relatives' testing in FH by evaluating the utility of a family sharing tool (FST) that includes a conversational agent, also known as a chatbot. Over 58% of individuals with FH consented to the FST. Genetic testing uptake was significantly greater among family members of those who consented to the FST compared to the relatives of FH patients who declined (8.8% vs. 19.0%, *p* < 0.001). The authors reported that the FST promoted more FH cascade testing than what has been reported in the literature (59% vs. 23%), demonstrating that electronic cascade testing tools can complement traditional approaches used by genetic counselors to assist with family communication (94). As new innovative tools become available, more medical centers and consumers may be able to readily access genetic counseling services.

### 6.3. Introducing advanced specialization models

**Figure 1:**
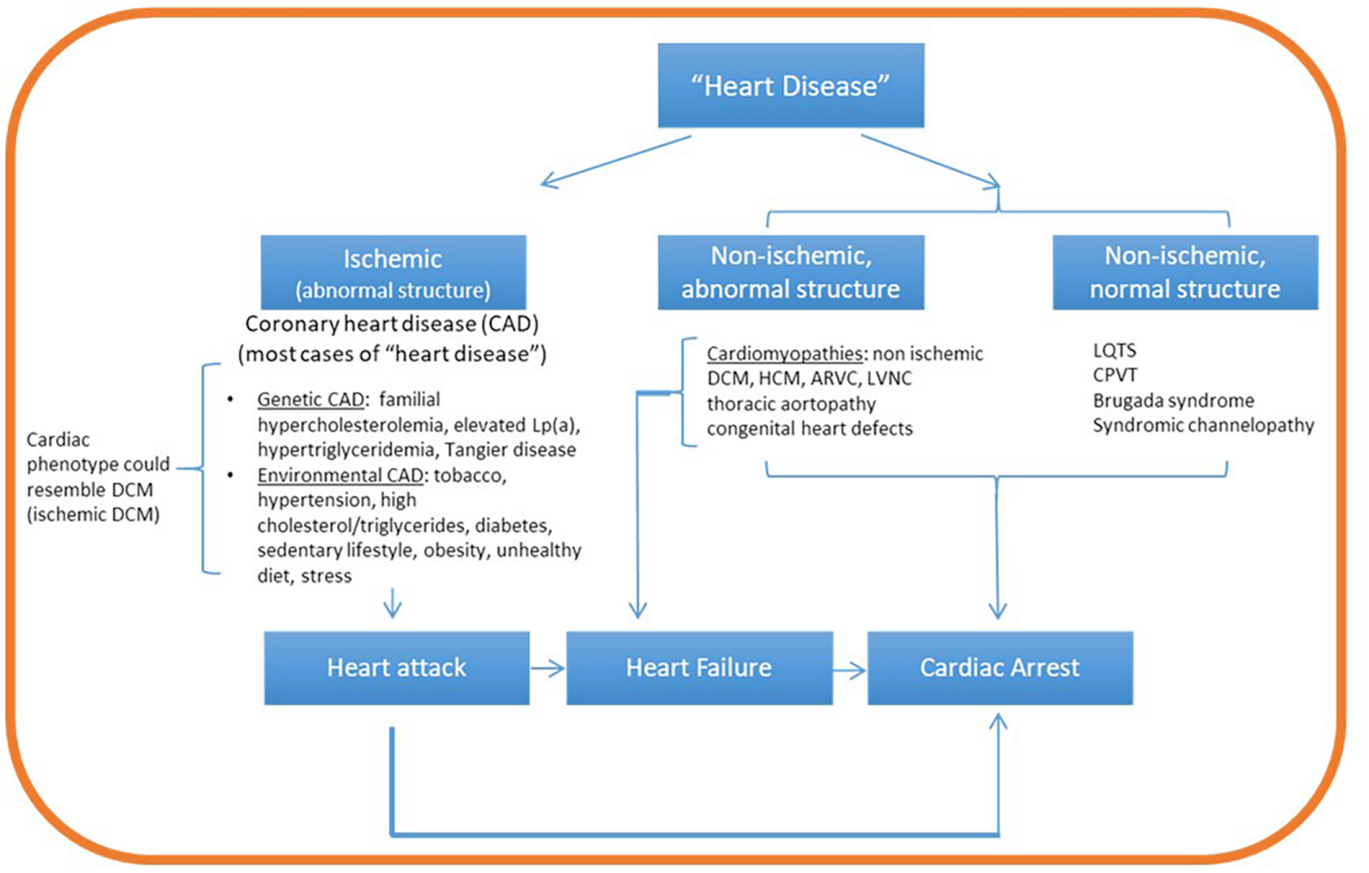
Conceptual map guiding a targeted path of inquiry in cardiovascular genetic counseling. Cardiovascular genetic disorders can be broadly classified as ischemic or non-ischemic. While ischemic disease is associated with structural abnormalities, non-ischemic heart disease can be associated with normal or abnormal heart structure. Oxygen deprivation from ischemic heart disease (coronary artery disease) can lead to myocardial death (infarction, heart attack) and cardiac arrest. Non ischemic structural disease (mainly the cardiomyopathies) can lead to heart failure and/or cardiac arrest. On the other hand, non-ischemic disease with normal structure (mainly channelopathies) lead directly to cardiac arrest. Family history questions and patient chart review guided by this framework can aim to identify evidence of ischemia (for example, coronary artery blockage from cardiac catheterization or coronary calcium scoring) and its potential role in disease etiology. If no evidence of ischemia is identified, proceed to identify evidence of structural disease (ventricular enlargement, aortic aneurysm or other defects from echocardiogram or magnetic resonance imaging) or arrhythmia (associated with normal structure) by ECG or Holter monitoring examination. Lp(a), lipoprotein A; DCM, dilated cardiomyopathy; HCM, hypertrophic cardiomyopathy; ARVC, arrhythmogenic right ventricular cardiomyopathy; LQTS, long QT syndrome.

The accelerating pace of developments in genetic research and clinical application renders imperative the lifelong learning for certified genetic counselors, as well as the establishment of genetic counseling subspecialties. Genetic assessment in the CVD clinic requires a detailed understanding of heritable CVDs and their potential overlap, patterns of inheritance, available clinical and research genetic testing, genetic variant interpretation, specialized pretest and posttest counseling, and cascade family testing for early detection (17, 95, 96). Furthermore, as population databases expand and encompass a wider range of ethnic and racial groups, variant reinterpretation requires an ongoing commitment. Basic understanding of fundamental cardiac anatomy, physiology and pathophysiology is also valuable. It therefore comes as no surprise that the voices advocating for the establishment of cardiovascular genetic counseling as a distinct subspecialty are multiplying (6, 64). While intuitively, it may seem as if practicing cardiovascular genetic counseling requires a sophisticated understanding of cardiac anatomy and physiology, cardiology-focused learning objectives for genetic counselors should be strategically outlined to facilitate the inquiry path of a genetic evaluation. For example, having a high-level understanding of the heart anatomy (e.g., major blood vessels, atria, and ventricles location), understanding the main activities of the heart (e.g., expansion, contraction, blood circulation, rhythm pace), and the most informative findings associated with genetic conditions should provide a roadmap for the genetic counselor to conduct a targeted chart review and medical and family history intake (Figure 1). Organizations such as the Accreditation Council for Genetic Counseling, the AHA, the American College of Cardiology Foundation, and the American Boards of Internal Medicine, Pediatrics, and Medical Genetics could play a leading role in developing standard curricula and establishing core competencies for trainees in cardiovascular genetic counseling.

While demonstrating an appropriate skill set may warrant certificate programs in cardiovascular genetic counseling, such programs should be carefully designed to avoid creating unintended barriers. For example, a new certificate requirement cannot create the unintended consequence of further exacerbating the genetic counseling shortage, which has been reported in the United States (75). Similarly, having a certificate in cardiogenetics should not impede an otherwise board-certified genetic counselor from practicing in a non-cardiology setting. In its mission to ensure minimal competence and monitor certification and recertification of genetic counselors, the American Board of Genetic Counseling (ABGC) has recognized the challenges associated with the rapidly evolving genetic counseling practice. Consequently, the ABGC Continued Competence for Genetic Counselors Taskforce was charged with making recommendations about the current recertification process for certified genetic counselors (personal communication, 2022). In this setting, genetic counseling recertification could include a demonstration of minimal competence in relevant areas not assessed in the genetic counselor's initial board exam, including, as appropriate, cardiogenetics items.

## 7. Conclusions

The combination of rapidly evolving genomics tools, knowledge and tests are gradually transforming the way Cardiology is practiced. Genetic risk assessment, specialized informed consent, genetic test selection, complex genetic test result interpretation (and reinterpretation), design and coordination of cascade testing, follow-up of patients with VUS, along with patient support at multiple levels, are only some of the skills needed in the Cardiology clinic of the Precision Medicine era. Specialized cardiovascular genetic counseling programs, expanded continuing education opportunities, revised training and certification procedures, curricula harmonization, along with innovative online services, telemedicine, and patient-facing digital tools appear to be the most effective way forward. The speed of implementation of these reforms will be of essence in the translation of scientific advancements into measurable benefits for CVD patients.

## Data Availability

The original contributions presented in the study are included in the article, further inquiries can be directed to the corresponding author.
